# Supplementary material for: Feasibility and performance of the fecal immunochemical test (FIT) for average-risk colorectal cancer screening in Nigeria
Source: PLoS One. 2021 Jan 12;16(1):e0243587. doi: 10.1371/journal.pone.0243587 (PMC7802943; doi:10.1371/journal.pone.0243587)
Supplement: S1 Table — (DOCX) [file pone.0243587.s001.docx]

S1 Table: Bivariate analysis for association between personal health history and FIT result

| **Covariate** | **FIT result** | | | | ***p* value** |
| --- | --- | --- | --- | --- | --- |
|  | Negative | | Positive | |  |
|  | *n* | % | *n* | % |  |
| **ASA or NSAID use within 7 days** | | | | | |
| No | 194 | 79.5 | 50 | 20.5 | 0.99 |
| Yes | 70 | 79.5 | 18 | 20.5 |  |
| **Heart burn / dyspepsia** | | | | | |
| No | 213 | 80.1 | 53 | 19.9 | 0.73 |
| Yes | 50 | 78.1 | 14 | 21.9 |  |
| **Diabetes mellitus** | | | | | |
| No | 244 | 79.2 | 64 | 20.8 | 0.80* |
| Yes | 20 | 83.3 | 4 | 16.7 |  |
| **Hypertension** |  |  |  |  |  |
| No | 200 | 81.0 | 47 | 19.0 | 0.26 |
| Yes | 64 | 75.3 | 21 | 24.7 |  |
| **Cigarette use** |  |  |  |  |  |
| No | 254 | 79.4 | 66 | 20.6 | 1.00* |
| Yes | 10 | 83.3 | 2 | 16.7 |  |
| **Change bowel habits** | | | | | |
| No | 248 | 79.7 | 63 | 20.3 | 0.78* |
| Yes | 16 | 76.2 | 5 | 23.8 |  |
